# Pancreatic tuberculosis with splenic tuberculosis mimicking advanced pancreatic cancer with splenic metastasizes: a case report

**DOI:** 10.1186/1757-1626-1-84

**Published:** 2008-08-12

**Authors:** YF Rong, WH Lou, DY Jin

**Affiliations:** 1 Pancreatic Group, Department of General surgery, Zhongshan Hospital, Fudan University, Shanghai, 200032, PR China

## Abstract

A 60-year-old woman presented with vague abdominal pain for one week was referred to pancreatic tail carcinoma accompanied with splenic metastasizes. She came to our hospital for further treatment. Ultrasonography and abdominal computed tomography (CT) revealed a pancreatic tail tumor with splenic metastasizes. There was no history of tuberculosis. Laparotomy was performed because pancreatic tail carcinoma with splenic metastasizes was highly suspected. Indurated mass in the pancreatic tail and sporadic metastasizes in the spleen had been found during the surgery. The pancreatic tail and the spleen were removed and proved to be tuberculosis on histological examination of a frozen section. The patient was given antituberculosis therapy and is now getting well. Tuberculosis should be considered in the differential diagnosis of pancreatic masses. The response to antituberculosis treatment is very favorable.

## Background

Pancreatic tuberculosis (TB) is considered to be extremely rare. However, in the past 20 years, the report of the pancreatic TB has increasing. Most cases of pancreatic TB are diagnosed only after tissue pathology via biopsy or exploratory laparotomy. Because almost all cases of pancreatic TB are effective to antituberculosis management, highlighted awareness of the incident of pancreatic tuberculosis could help to manage this disease. Here we present a case of TB in the pancreatic tail and spleen mimicking pancreatic carcinoma with splenic metastasizes in a 60-year-old woman from China.

## Case representation

The patient was a 60-year-old woman. She had a past medical history significant for appendectomy, cholecystectomy, left ovariotomy, left breast fibromectomy, left parotectomy and lung nodal with the therapy of steroid. She initially went to the outside hospital on Jan 22, 2007, with complaints of upper abdominal pain. She described her pain as nonconstant and variable in severity. She denied weight loss, fever, chills, nausea, or vomiting. Her physical examination at the time was unremarkable and included normal respiratory, cardiovascular, and abdominal examinations. Her routine laboratory test values were normal, including liver function tests and tumor mark tests. Abdominal ultrasonography scan showed that sporadic metastasizes in the spleen and the common computed tomography (CT) scan showed the malignant tumor in the tail of the pancreas with metastasizes in the spleen.

**Figure 1:**
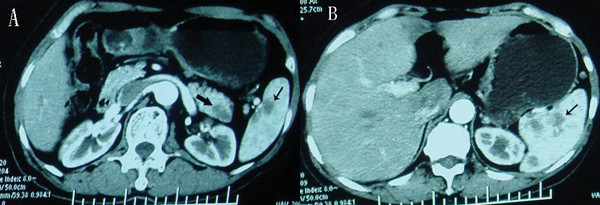
**CT scans of the pancreas**. CT scan of pancrease demonstrating a mass in the pancreatic tail () and metastasizes in the spleen (→).

**Figure 2:**
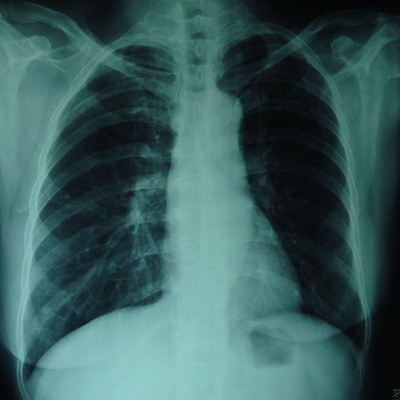
**Chest X-ray**. Chest X- ray shows no TB signs.

She was referred to our department for surgical treament on Jan 25, 2007. At this time, her symptoms and physical examination results remained unchanged. Hemoglobin: 127 g/dL, white blood cell count: 4,300/mm^3^, platelets: 147 × 10^3^/mm^3^, AST: 26 U/L, ALT: 15 U/L, total bilirubin: 14.3 μmol/L, serum creatinine: 76 μmol/L, CEA: 1.29 ng/dL, CA 199:7.1 U/L (normal < 37 U/L). HIV test was negative. A CT scan (performed as a three-dimensional multidetector scan) revealed a low-density mass in the tail of the pancreas with metastasizes in the spleen (Fig 1A and 1B). There was no evidence of ascites, and all of the perihepatic and peripancreatic visceral vessels were not invaded. And the Chest X-ray had no positive founding (Fig 2). Exploratory laparotomy was performed because pancreatic tail carcinoma with splenic metastasizes was highly suspected.

At abdominal exploration on Jan 29, 2007, there were multiple nodules present in the pancreatic tail and spleen (with the diameter of 0.5–2 cm). So we excised the pancreatic tail and the spleen. Once the specimen had been removed, it was submitted to histological examination of a frozen section. The specimen sent to pathology revealed granulomas with caseating necrosis with Langhan's giant cells, suspicious for TB. Given the information from frozen section, the decision was made to close the abdomen without lymph nodes clearance.

**Figure 3:**
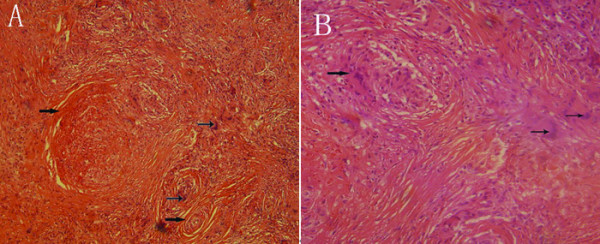
**(A and B) Histological examination**. Fig 3A low power view of the pancreas demonstrating granulomatous inflammation () and Langhans' giant cells (→) (H & E stain, 10×); Fig 3B high power view of the same field of Fig 3A (H & E stain, 20×).

All pathology specimens were routinely processed (10% formalin), paraffin embedded, and stained with hematoxylin and eosin. Histopathologic examination demonstrated multiple granulomas with caseating necrosis, highly suspicious for TB (Fig 3A and 3B).

After the operation, the patient was placed in appropriate isolation with strict contact and droplet precautions. The postoperative course was uncomplicated, and the patient was discharged on postoperative day 7 in stable condition. Before her discharge, her case was reported to the Shanghai Health Department and send to the lazaretto for the further therapy of the TB. She has been followed and is currently doing well, having completed her anti-TB therapy.

## Discussion

TB is caused by the pathogen *M. tuberculosis*, a rod-shaped aerobic bacterium notable for its acid-fast staining properties which is still a common disease worldwide. The incidence, prevalence, and mortality of the disease vary greatly among different nations, and notably all forms of TB are continuing to increase in all regions [1]. Possible explanations include increased cases of HIV, expanded use of immunosuppressant therapy, globalization of the world's population, and increased transmission in environments such as prisons, homeless shelters, other reasons like evolutionary changes in the biology of the bacterium, drug resistance and so on[2].

Although TB often occurs in lung, primary abdominal TB is not uncommon. In 1986, for example, of the 22,768 reported cases of TB in the United States, only 0.58% was found to be located in the peritoneum [3]. However the prevalence of abdominal TB in developing countries has been estimated to be as high as 12% [4]. Although one might expect abdominal TB to be accompanied with active pulmonary TB, only 6–38% had this association [5]. TB does easily disseminate to the gastrointestinal tract, liver, spleen and mesenteric lymph nodes; however the involvement of pancreatic TB is rare. In 1944 Auerbach first reported TB mimicking pancreatic cancer [6]. He described 1656 autopsies of tuberculous patients and identified 297 cases of miliary TB. Only 14 cases had direct pancreatic involvement that may have mimicked neoplasia. Franco-Paredes and colleagues reported two cases of pancreatic TB and reviewed the current literature involving pancreatic TB among nonimmunosuppressed individuals [7]. The authors found between 1980 and 2002 that 50 cases of pancreatic TB had been reported. Thirteen of these cases were categorized as pancreatic masses, mimicking pancreatic carcinoma. While Eric S reported an additional 25 cases of pancreatic TB presenting as discrete pancreatic masses and 42 cases of peripancreatic TB mimicking pancreatic masses in nonimmunosuppressed individuals [2]. In Chinese language literature there were more than 70 cases of pancreatic TB having been described [8-14].

The diagnosis of pancreatic TB often proves to be extremely challenging. This is in part because the presentation of abdominal TB is slow and insidious, with nonspecific signs and symptoms. In the present case, for example, vague upper abdominal pain was the only presenting symptoms. Those with immunosuppression status such as HIV positive patients or who live in endemic areas are more likely to develop pancreatic and peripancreatic TB. However in the absence of either of these characteristics, it would be difficult to identify patients with pancreatic TB. In China Feng Xia et al. reviewed literatures and revealed several clinical characteristics of pancreatic TB as follows:1) pancreatic TB is mostly suffered in young people, especially female, while pancreatic tumor is most common in old person; 2) some patients have a history of TB in past, and most often come from areas having high incidence of active tuberculosis; 3) the patients often present with epigastric pain, fever and weight loss; 4) ultrasound and CT scan show pancreatic mass and peripancreatic nodules, some with focal calcification[8].

Abdominal TB presents with nonspecific signs and symptoms, making reliance on symptomatology alone for diagnosis insufficient. The diagnostic techniques used for pancreatic TB can be divided into two types: noninvasive and invasive. Noninvasive techniques rely mainly on CT. Sinan et al. reviewed the CT characteristics of pancreatic TB[15]. The most common features were peritoneal involvement and lymphadenopathy. Pancreatic involvement typically appears as an enhancing hypodensity mass, with irregular borders, occasionally mimicking pancreatic carcinoma. In contrast to noninvasive techniques, invasive diagnostic techniques can be used to obtain tissue for pathologic examination and thus are more reliable diagnostic tools. Techniques for biopsy include endoscopic US-guided biopsy, CT/US-guided percutaneous biopsy, and surgical biopsy (open or laparoscopic). Unfortunately, in most cases in the literature, the diagnosis of peripancreatic TB was made only after exploratory laparotomy, as in the present case.

For a definitive diagnosis of peripancreatic TB, microbiological and/or histologic confirmation is needed. In the review of Eric S and colleagues suggested that direct smear for the detection of acid-fast organisms is less sensible than combined visual and histopathologic diagnosis [2]. Once the tissue diagnosis has been made, the management of TB rest on the medical treatment. Medical management of pancreatic TB generally consists of isoniazid and rifampin, with pyrazinamide and ethambutol added for severe or resistant cases. In addition to anti-TB treatment, prevention of bacterium spread to other individuals is essential in the management of the disease. While in the hospital, the patient must be quarantined and hospital staff must wear appropriate protective clothing to protect themselves and prevent spread.

## Conclusion

The present case illustrates pancreatic tail and splenic TB mimicking pancreatic malignant with splenic metastasizes. Once the correct diagnosis was made, our patient was started on a four-drug regimen of anti-TB drugs. However the diagnosis of pancreatic TB often is extremely difficult. This is because patients present with nonspecific symptoms and signs. In addition, CT scan does not reliably distinguish pancreatic TB from pancreatic adenocarcinoma, especially the current case with splenic TB. In present patient we did not pay much attention to the history of taking steroid with an immunosuppressed status. In conclusion, pancreatic TB is rare and the diagnosis is challenging. However if doctors are aware of its clinical features and conduct more diagnostic modalities including CT scan and ultrasound-guided FNA or laparoscopic biopsy, diagnosis of pancreatic tuberculosis without laparotomy is possible and the disease can be effectively treated with antituberculous drugs.

## Competing interests

The authors declare that they have no competing interests.

## Authors' contributions

YFR was a major contributor in writing the manuscript. WHL and DYJ performed the surgery and give consultation to the manuscript. All authors read and approved the final manuscript.

## Consent section

Written informed consent was obtained from the patient for publication of this case report and accompanying images. A copy of the written consent is available for review by the Editor-in-Chief of this journal.
